# Predictors of responders to mononuclear stem cell-based therapeutic angiogenesis for no-option critical limb ischemia

**DOI:** 10.1186/s13287-018-1117-5

**Published:** 2019-01-11

**Authors:** Tianyue Pan, Hao Liu, Yuan Fang, Zheng Wei, Shiyang Gu, Gang Fang, Yifan Liu, Yang Luo, Daqiao Guo, Xin Xu, Bin Chen, Junhao Jiang, Jue Yang, Zhenyu Shi, Ting Zhu, Yun Shi, Peng Liu, Zhihui Dong, Weiguo Fu

**Affiliations:** 1 Department of Vascular Surgery, Zhongshan Hospital, Fudan University, Shanghai, 200032 China; 2 Institute of Vascular Surgery, Fudan University, Shanghai, China; 3 Department of Hematology, Zhongshan Hospital, Fudan University, Shanghai, China

**Keywords:** Critical limb ischemia, Therapeutic angiogenesis, Mononuclear cell transplantation, Predictive analysis, Nomogram

## Abstract

**Background:**

Although the mononuclear cell (MNC) transplantation could theoretically induce therapeutic angiogenesis in the patients with no-option critical limb ischemia (NO-CLI), the clinical responses to this approach are inconsistent among different clinical trials. The purpose of this study was to identify the prognostic factors of responders and develop a predictive nomogram to guide patient selection.

**Methods:**

We retrospectively reviewed a consecutive NO-CLI cohort who received peripheral blood-derived transplantation in our center. The patients who survived and achieved complete remission of CLI at 6 months post-transplantation were defined as responders. Logistic regression models were used to screen and identify the prognostic factors based on which predictive nomogram was developed. A receiver operating characteristic (ROC) curve and a calibration curve were drawn to determine the discrimination level and predictive accuracy.

**Results:**

The study ultimately enrolled 103 patients, including 58 responders and 45 non-responders. Based on the multivariate regression analysis, age ≥ 50 years (odds ratio [OR] 0.201, *P* = 0.004), blood fibrinogen > 4 g/L (OR 0.176, *P* = 0.003), arterial occlusion above the knee/elbow (OR 0.232, *P* = 0.010), the transcutaneous pressure of oxygen (TcPO_2_) (OR 1.062, *P* = 0.006), and the Log total transplanted CD34^+^ cell count (OR 3.506, *P* = 0.046) were identified as independent prognostic factors of the responders in the nomogram. An area under the ROC curve of 0.851 indicated good discrimination, and the calibration curve of the predicted probability showed optimal agreement with that of the observed probability.

**Conclusions:**

Age, blood fibrinogen, arterial occlusion level, TcPO_2_, and the total transplanted CD34^+^ cell count were independent prognostic factors of the responders. A nomogram with high discrimination and accuracy was developed to provide individualized predictions.

**Trail registration:**

ChiCTR, ChiCTR1800019401. Registered 9 November 2018—Retrospectively registered

**Electronic supplementary material:**

The online version of this article (10.1186/s13287-018-1117-5) contains supplementary material, which is available to authorized users.

## Background

As the terminal stage of peripheral artery disease (PAD), critical limb ischemia (CLI), which is characterized by rest pain and unhealing wounds of the limbs, is associated with a 1-year major amputation rate of 30% and mortality rate of 25% [1, 2]. Currently, endovascular and surgical revascularizations are the effective mainstream interventional strategies for CLI [3]. However, up to 30% of CLI patients are not suitable candidates for these procedures due to distal runoff deficiency or rapid postoperative arterial re-occlusion [4]. In these so-called no-option CLI (NO-CLI) patients, the prognosis appears to be even worse, with a major amputation rate of 40% at 6 months from onset [2]. Novel approaches of stem cell-based therapeutic angiogenesis, including bone marrow- (BM) or peripheral blood (PB)-derived mononuclear cell (MNC) transplantation with or without purification of specific cell types, are increasingly being used in clinical trials that attempt to treat NO-CLI [5–13]. These phase I/II trials have confirmed the safety and feasibility of MNC transplantation and revealed its potential therapeutic benefits. However, the curative effects are inconsistent among different study populations. Several clinical trials have shown the positive therapeutic efficacy of MNC or purified CD34^+^ cell transplantation in treating NO-CLI patients with respect to avoiding major amputations and promoting wound healing [12, 14, 15]. In contrast, other trials have observed an insignificant moderate prognosis following such therapeutic approaches relative to conservative treatments or placebo [7, 11]. These controversial results might be attributed largely to the considerable heterogeneity of the study population. Younger patients with thromboangiitis obliterans (TAO) have been shown to achieve more therapeutic benefits than older patients with arteriosclerosis obliterans (ASO) [11, 16]. Notably, aging was considered to be a critical factor for attenuating blood perfusion restoration after cell transplantation [17]. In addition, the concomitant cardiovascular diseases and organ dysfunction would probably lower the survival rate of the elderly PAD/CLI population, thereby compromising the therapeutic benefits of the MNC treatment. Another important factor relating to prognosis is the feature of the transplant. Although the therapeutic mechanism of mononuclear cells remains to be clarified, the dosage of transplanted CD34^+^ cells is regarded as a key factor for ineffective revascularization and blood supply restoration [18]. Meta-analyses have revealed that in clinical trials, patients do not respond favorably to MNC transplantation with a relatively low dosage of CD34^+^cells or MNCs [19, 20].

To date, the characteristics of the patients who benefit from MNC transplantation have not been comprehensively investigated. Due to the conflicting findings of different clinical trials, the patient selection criterion of this approach should be reexamined and clarified. This study was performed to explore the clinical background features and transplants of both responders and non-responders to MNC transplantation and to develop a nomogram to practically formulate an individualized prognostic prediction and clarify the criterion.

## Methods

### Patients

A consecutive cohort from May 2009 to October 2017 was retrospectively enrolled in our center and included 110 patients with NO-CLI who received peripheral blood mononuclear cell (PBMNC) transplantation with or without purification for CD34^+^ cells. The patients included in the study met the following inclusion criteria: (1) provision of signed informed consent, (2) male or female aged between 18 and 80 years, (3) diagnosis of limb ischemia due to arterial stenosis or occlusion, as confirmed by computed tomographic angiography, magnetic resonance angiography, or digital subtraction angiography, (4) rest pain or tissue loss under the superior level of metatarsus (Rutherford class 4 or 5) that was anatomically unsuitable for surgery or an endovascular intervention or showing no improvement for at least 3 months following surgery or an endovascular intervention, (5) if present, unrelieved rest pain or a wound size that was not reduced after at least 1 month of medical treatment or other conservative treatment including smoking cessation, dietary control, and exercise therapy, and (6) complete baseline data and known status of survival, amputation, wound healing, and rest pain relief at 6 months after transplantation. The exclusion criteria were (1) serious health events < 3 months before admission, including but not limited to a myocardial infarction, cerebral apoplexy, a pulmonary embolism, and severe hepatic and renal dysfunction, (2) a diagnosis or suspicion of malignancy at baseline, or (3) a life expectancy < 6 months.

This study was approved by the ethics committee of Zhongshan Hospital, Fudan University, China. It was performed in agreement with the ethical principles of the Declaration of Helsinki.

### Procedures for cell transplantation

All the patients received subcutaneous injections of rhG-CSF (Neupogen®; Amgen, Thousand Oaks, CA, USA) (5–10 μg/kg per day for 4 days) to mobilize the BM cells, and enoxaparin (4000 IU/day) was administered daily to prevent hypercoagulable states. On the fifth day, a suspension of PBMNCs (200 mL) was collected via leukapheresis (COM.TEC; Fresenius Hemocare GmbH, Bad Homburg, Germany). The non-purified PBMNC transplants were obtained by washing three times and resuspending the apheresis products in an ethylenediaminetetra-acetic acid-phosphate buffered saline solution (200 mL) that contained 0.5% human albumin. The purified CD34^+^ cell transplants (40 ml) were obtained using a magnetic cell sorting system (MiltenyiBiotec GmbH, BergischGladbach, Germany) immediately after resuspension. The CD34^+^ cell counts of all the transplants were determined by leukocyte counting and flow cytometry using CD34 antibody. The final implanted CD34+ cells was set at a dosage ranging from 10^5^ to 10^6^ per kilogram body weight and the cells were transplanted into the ischemic limbs via equidistant intramuscular injections (0.5 mL/site) under general anesthesia. The injections were distributed in the calves/forearms and feet/hands in the patients with arterial occlusions above the ankle/wrist. In those with occlusions below the ankle/wrist, the injections were only administered in the feet/hands.

### Baseline information and endpoints

The baseline information was collected from the medical record of each patient, and this information included the demographic data, the etiology of the limb ischemia, the risk factors for peripheral artery and cardiovascular diseases, the critical results of blood examination, and the treatment history. The baseline features of the treated limb were also recorded, including the Rutherford class, the wound type, the ankle-brachial index (ABI), the transcutaneous pressure of oxygen (TcPO_2_) of the dorsum, and the occlusion level of the arteries according to the pretherapy radiological imaging. The primary endpoint was CLI remission, defined as surviving patients with complete wound healing and rest pain relief, without major limb amputation at 6 months after transplantation. The patients who achieved CLI remission were regarded as responders. The other patients were regarded as non-responders. The secondary endpoints included mortality and the major amputation-free survival rate at 6 months.

### Statistical analysis

The baseline characteristics of the responders and non-responders were compared first. Continuous variables were presented as the means ± standard deviations (SDs) or as the medians ± interquartile ranges (IQRs) according to the data distribution. The independent Student’s *t* test or Mann-Whitney *U* test was used to analyze the significance of the differences between responders and non-responders. A paired *t*-test or Wilcoxon signed-rank test was used to analyze the longitudinal changes from baseline to 6 months post-transplantation. Categorical variables were presented as numbers with percentages, and the Pearson chi-square test or Fisher’s exact test was used to analyze the significance of the differences. Subsequently, the candidate prognostic factors of the responders were screened through a univariate binary logistic regression analysis. The correlations between the candidate predictors or the variables at different time points were analyzed with linear regression or Pearson’s chi-square test. Furthermore, a multivariate binary logistic regression model was used to identify the independent predictors. Accordingly, a nomogram was developed, followed by performance of the receiver operating characteristic (ROC) curve with the area under the curve being used to evaluate the discrimination level. The calibration curve, which was adjusted with 1000 cycles of bootstrap resampling, was used to analyze the agreement between the predicted and the observed probability of the responders. A two-tailed *P* value < 0.05 was considered statistically significant. The statistical analyses were performed with SPSS version 19.0 for Windows (SPSS, Chicago, IL, USA), and the nomogram and the calibration curve were drawn using R software (version 3.4.3) with rms package (http://www.r-project.org).

## Results

### Baseline information and endpoints

**Fig. 1:**
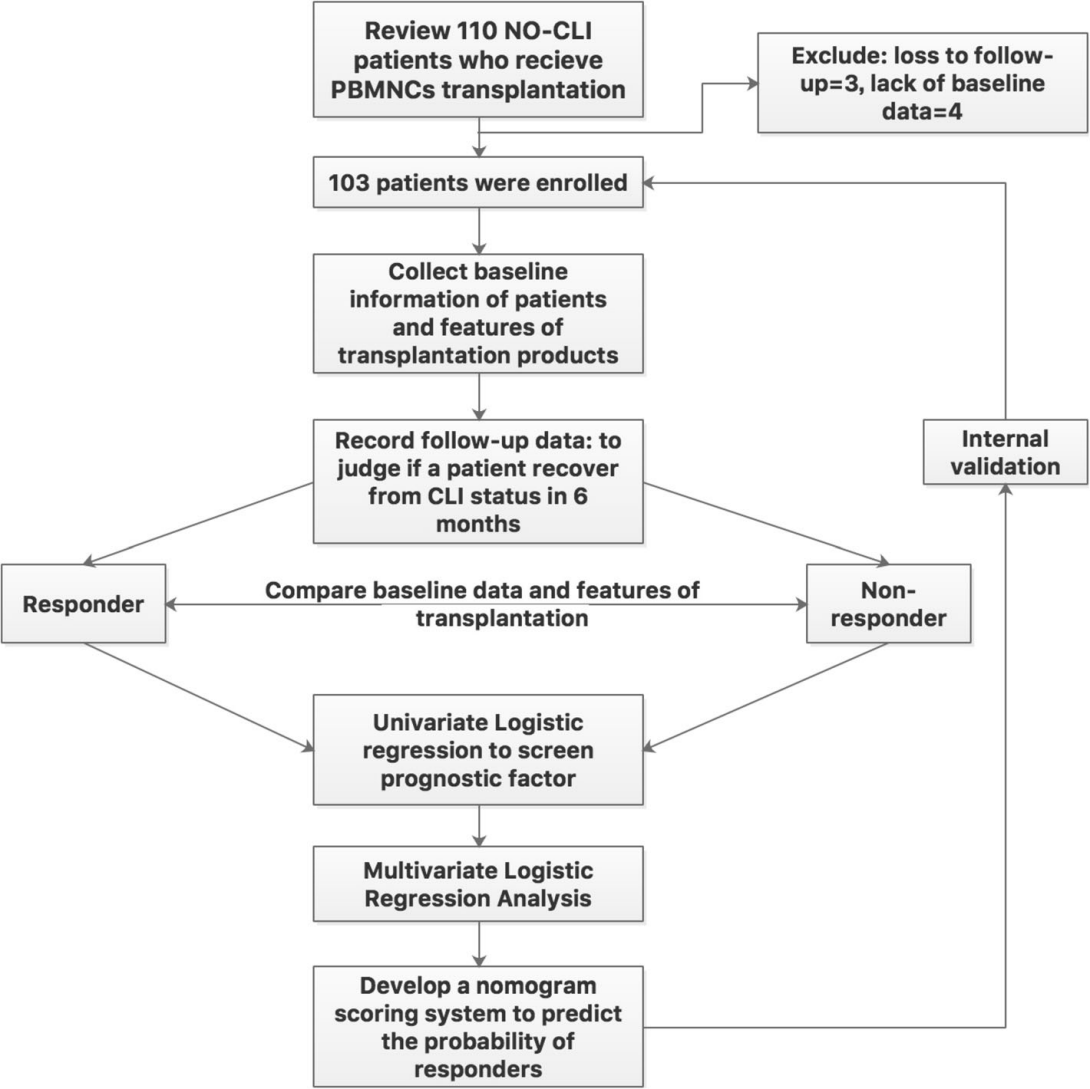
Study flow diagram. NO-CLI, no-option critical limb ischemia

Of the 110 patients reviewed, 3 were lost to follow-up within 6 months, and the critical baseline information of the other 4 was lacking. Therefore, 103 patients with complete data at the 6-month follow-up were finally included and analyzed (Fig. 1). The mean age of the patients was 44.9 ± 13.1 years, and the male ratio was 98.1%. The etiologies included TAO (86 patients), ASO (10 patients), collagen disease (4 patients), Crohn’s disease (1 patient), and eosinophilia (2 patients) (Table 1). There were 99 lower limbs and 4 upper limbs involved in the study. The baseline Rutherford class was 5 in 91 patients (88.4%) and 4 in 12 patients (11.6%) (Table 1). The median total CD34^+^ cell count in the transplants was 43.0 × 10^6^, with an IQR of 26.0–84.0 × 10^6^. The 6-month major amputation-free survival rate was 92.2% (95/103), wherein 3 patients with TAO and 4 patients with ASO underwent major amputations (6 above-the-knee cases and 1 below-the-knee case) and 1 patient with TAO died at 2 months due to hepatic failure of unknown cause. A total of 56.3% (58/103) patients achieved complete remission from CLI at 6 months and were regarded as responders to the MNC transplantation (Table 1).

**Table 1:** Baseline characteristics of patients

	Total, *n* = 103	Responders, *n* = 58	Non-responders, *n* = 45	*P* value
Age (years) (mean ± SD)	44.9 ± 13.1	43.4 ± 11.2	46.8 ± 15.2	0.204
20–29, *n* (%)	16(15.5%)	8(13.8%)	8 (17.8%)	0.596
30–39, *n* (%)	18 (17.5%)	13 (22.4%)	5 (11.1%)	0.191
40–49, *n* (%)	33 (32.0%)	24 (41.4%)	9 (20.0%)	0.033
50–59, *n* (%)	25 (24.3%)	10 (17.2%)	15 (33.3%)	0.068
60–69, *n* (%)	4 (3.9%)	0 (0.0%)	4 (8.9%)	0.034
70–79, *n* (%)	7 (6.8%)	3 (5.2%)	4 (8.9%)	0.696
< 50, *n* (%)	67 (65.0%)	45 (77.6%)	22 (48.9%)	0.002
≥ 50, *n* (%)	36 (35.0%)	13 (22.4%)	23 (51.1%)	0.002
Gender				
Male, *n* (%)	101 (98.1%)	56 (96.6%)	45 (100.0%)	0.208
Female, *n* (%)	2 (1.9%)	2 (3.4%)	0 (0.0%)	0.208
Body mass index (kg/m^2^) (mean ± SD)	23.3 ± 3.1	23.4 ± 3.0	23.1 ± 3.3	0.630
Etiology of limb ischemia				
TAO, *n* (%)	86 (83.5%)	52 (89.7%)	34 (75.6%)	0.056
ASO, *n* (%)	10 (9.7%)	3 (5.2%)	7 (12.1%)	0.078
Collagen disease, *n* (%)	4 (3.9%)	2 (3.4%)	2 (4.4%)	0.795
Crohn’s disease, *n* (%)	1 (1.0%)	1 (1.7%)	0 (0.0%)	0.856
Eosinophilia, *n* (%)	2 (1.9%)	0 (0.0%)	2 (4.4%)	0.105
Risk factors of cardiovascular disease				
Hypertension, *n* (%)	7 (6.8%)	2 (3.4%)	5 (11.1%)	0.125
Diabetes mellitus, *n* (%)	7 (6.8%)	2 (3.4%)	5 (11.1%)	0.125
Hyperlipidemia, *n* (%)	5 (4.9%)	4 (6.9%)	1 (2.2%)	0.274
Smoker, *n* (%)	85 (82.5%)	51 (87.8%)	34 (75.6%)	0.101
Blood examination				
Fibrinogen, (mg/dL) (median [IQR])	316.0 (255.0–408.0)	304.5 (238.0–351.0)	336.0 (278.0–467.0)	0.012
CRP, (mg/L) (median [IQR])	5.40 (1.90–23.00)	3.75 (1.60–8.50)	9.50 (4.00–40.80)	0.001
Glucose, (mmol/L) (median [IQR])	4.70 (4.40–5.00)	4.70 (4.50–5.00)	4.70 (4.40–5.00)	0.762
Creatinine, (μmol/L) (median [IQR])	72.0 (64.0–83.0)	73.0 (67.0–83.0)	70.0 (63.0–76.0)	0.102
Treatment history				
Antiplatelet drugs, *n* (%)	58 (56.3%)	34 (58.6%)	24 (53.3%)	0.592
Vasodilator, *n* (%)	55 (53.4%)	33 (56.9%)	22 (48.9%)	0.419
Statins, *n* (%)	4 (3.9%)	2 (3.4%)	2 (4.4%)	0.795
Stenting, *n* (%)	10 (9.7%)	5 (8.6%)	5 (11.1%)	0.672
Balloon dilation, *n* (%)	7 (6.8%)	4 (6.9%)	3 (6.7%)	0.963
Thrombolysis, *n* (%)	16(15.5%)	8(13.8%)	8 (17.8%)	0.580
Thrombectomy, *n* (%)	8 (7.8%)	3 (5.2%)	5 (11.1%)	0.264
Bypass surgery, *n* (%)	5 (4.9%)	2 (3.4%)	3 (6.7%)	0.451
Endarterectomy, *n* (%)	1 (1.0%)	1 (1.7%)	0 (0.0%)	0.376

SD, standard deviation; IQR, interquartile range; TAO, thromboangiitis obliterans; ASO, atherosclerosis obliterans; CRP, C-reaction protein

### Characteristics of responders and non-responders

The mean age of the responders tended to be lower than that of the non-responders, though the difference was not significant (43.4 ± 11.2 versus 46.8 ± 15.2 years, *P* = 0.204). However, the frequency of the patients whose ages were < 50 years in the responder group was significantly higher than that in the non-responder group (77.6% versus 48.9%, *P* = 0.002) (Table 1). Similarly, comparing frequency distribution trends of age in the responders and non-responders, an intersection point was observed at 50 (Additional file 1). When the baseline blood examinations were evaluated, the responders had significantly lower blood fibrinogen and CRP levels than the non-responders (Table 1). No significant differences were observed between the responders and non-responders in terms of the etiologies, risk factors of cardiovascular disease, or treatment histories (Table 1).

**Fig. 2:**
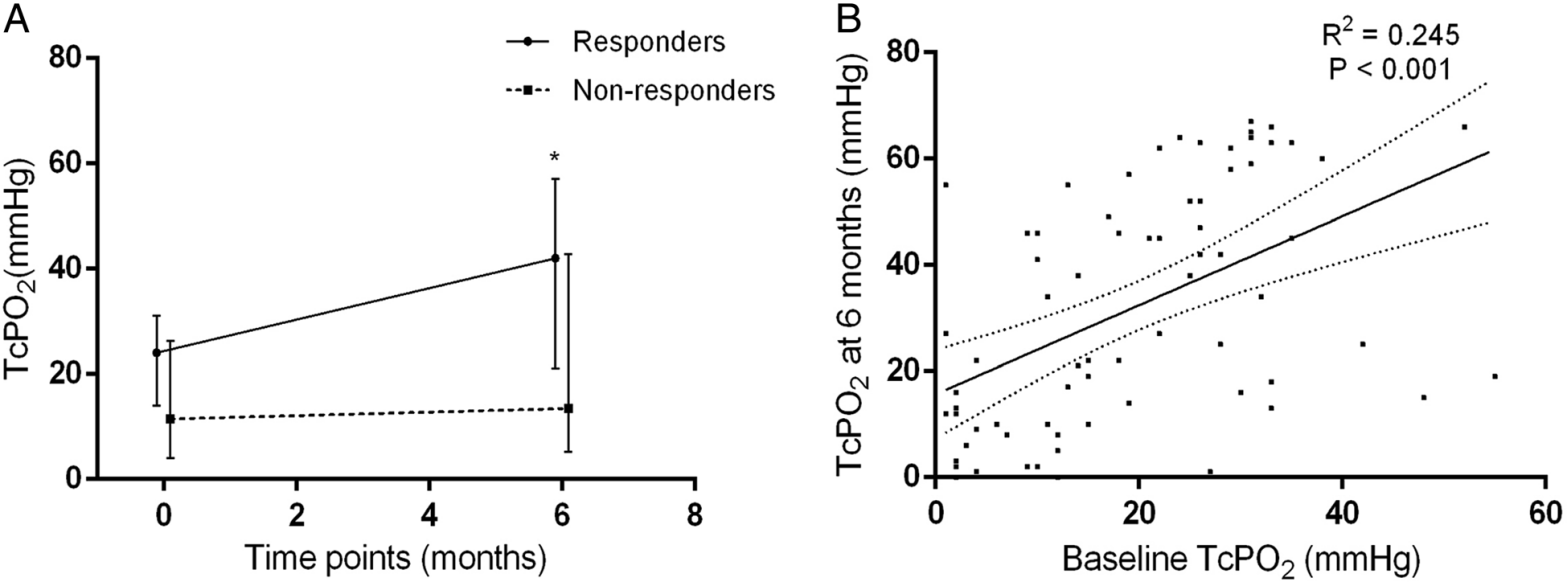
Comparison of longitudinal TcPO_2_ changes (median with interquartile range) in the ischemic limbs of the responders and non-responders (**a**) and linear regression between TcPO_2_ at baseline and at 6 months post-transplantation, depicted with a solid fitting line and dotted 95% confidential interval bars (**b**). *The difference between 6 months and baseline was statistically significant (*P* < 0.05). TcPO_2_, transcutaneous pressure of oxygen

Regarding the treated limbs, the responders were characterized by significantly higher baseline ABI (median 0.515, IQR 0.410–0.695) and TcPO_2_ (median 24 mmHg, IQR 14–30 mmHg) than the non-responders (ABI, median 0.430, IQR 0.280–0.540, *P* = 0.011; TcPO_2_, median 9 mmHg, IQR 4–19 mmHg, *P* < 0.001) (Table 2). Based on the available complete longitudinal data (*n* = 69), the median TcPO_2_ of the responders increased from 24 mmHg (IQR 14–31 mmHg) at baseline to 42 mmHg (IQR 21–57 mmHg) at 6 months post-transplantation (*P* < 0.001), while that of the non-responders did not improve significantly, increasing from 11.5 mmHg (IQR 4–26 mmHg) at baseline to 13.5 mmHg (IQR 5–43 mmHg) at 6 months post-transplantation (*P* = 0.094) (Fig. 2a). TcPO_2_ at baseline correlated significantly with that at 6 months (*R*^2^ = 0.245, *P* < 0.001) (Fig. 2b). Considering the aspect of arterial lesions, 60.02% of the patients were characterized by an occlusion level above the knee or elbow, including 3.9% in the common or external iliac artery, 4.8% in the common femoral artery or axillary artery, 35.0% in the superficial femoral artery or brachial artery, and 16.5% in the popliteal artery. The remaining 39.8% of the patients were characterized by a highest occlusion level below the knee, with 27.2% of occlusions located in the calves or forearms and 12.6% of occlusions located below the ankles or wrists. The responders had a significantly lower frequency of arterial occlusions above the knee or elbow than the non-responders (46.6% versus 77.8%, *P* = 0.001) (Table 2). In addition, no differences were observed between the responders and non-responders in terms of the Rutherford class (*P* = 0.277) or gangrene (*P* = 0.718) (Table 2). Regarding the transplants, the responders were not associated with preconditioning of CD34^+^ cell purification (*P* = 0.775) (Table 2). The total transplanted CD34^+^ cell count appeared to be higher in the responder group (median 51.5 × 10^6^, IQR 26.0–103.0 × 10^6^) than in the non-responder group (median 40.0 × 10^6^, IQR 26.0–68.0 × 10^6^), although the difference was not statistically significant (*P* = 0.114) (Table 2).

**Table 2:** Baseline characteristics of treated limbs and transplants

	Total, *n* = 103	Responders, *n* = 58	Non-responders, *n* = 45	*P* value
Rutherford class, *n* (%)				
IV	12 (11.6%)	5 (8.6%)	7 (15.6%)	0.277
V	91 (88.4%)	53 (91.4%)	38 (84.4%)	0.277
Gangrene, *n* (%)				
Yes	46 (44.7%)	25 (43.1%)	21 (46.7%)	0.718
No	57 (55.3%)	33 (56.9%)	24 (53.3%)	0.718
ABI* (median [IQR])	0.485 (0.370–0.640)	0.515 (0.410–0.695)	0.430 (0.280–0.540)	0.011
TcPO_2_ (mmHg) (median [IQR])	17 (6–28)	24 (14–30)	9 (4–19)	< 0.001
Highest level of arterial occlusion				
Above the knee or elbow, *n* (%)	62 (60.2%)	28 (46.6%)	34 (77.8%)	0.001
Common or external iliac artery, *n* (%)	4 (3.9%)	1 (1.7%)	3 (6.7%)	0.198
Common femoral artery or axillary artery, *n* (%)	5 (4.8%)	4 (6.9%)	1 (2.2%)	0.383
Superficial femoral artery or brachial artery, *n* (%)	36 (35.0%)	15 (25.9%)	21 (46.7%)	0.037
Popliteal artery, *n* (%)	17 (16.5%)	7 (12.1%)	10 (22.2%)	0.169
Below the knee or elbow, *n* (%)	41 (39.8%)	31 (53.4%)	10 (22.2%)	0.001
Artery in the calf or forearm, *n* (%)	28 (27.2%)	23 (39.7%)	5 (11.1%)	0.001
Artery below the ankle or wrist, *n* (%)	13 (12.6%)	8(13.7%)	5 (11.1%)	0.684
Type of transplantation				
Purified for CD34^+^ cells, *n* (%)	52 (50.5%)	30 (51.7%)	22 (48.9%)	0.775
Not purified, *n* (%)	51 (49.5%)	28 (48.3%)	23 (51.1%)	0.775
Total transplanted CD34+ cells (million/L) (median [IQR])	43.0 (26.0–84.0)	51.5 (26.0–103.0)	40.0 (26.0–68.0)	0.114
Cell viability (median [IQR])	98.4% (97.7%–98.6%)	98.3% (97.5%–98.6%)	98.4% (98.2%–98.6%)	0.212

*ABIs of 99 patients with lower limbs treated were included in this analysis, while the other 4 patients with upper limbs treated were excluded

IQR, interquartile range; ABI, ankle-brachial index; TcPO_2_: transcutaneous pressure of oxygen; CD, cluster of differentiation

### Prognostic factors

According to the results of the univariate logistic regression, several variables were screened out: age ≥ 50 years [odds ratio (OR) 0.276, 95% confidence interval (CI) 0.118–0.647, *P* = 0.003], CRP > 3 mg/L (OR 0.287, 95% CI 0.117–0.702, *P* = 0.006), fibrinogen > 4 g/L (OR 0.172, 95% CI 0.064–0.459, *P* < 0.001), ASO (OR 0.296, 95% CI 0.072–1.218, *P* = 0.092), highest arterial occlusion level above the knee or elbow (OR 0.249, 95% CI 0.104–0.595, *P* = 0.002), ABI (OR 8.345, 95% CI 1.301–53.54, *P* = 0.025), TcPO_2_ (OR 1.069, 95% CI1.030–1.109, *P* < 0.001), and base-10 logarithm (Log) of total transplanted CD34^+^ cell count (OR 2.274, 95% CI 0.850–6.079, *P* = 0.102) (Table 3). The linear regression analysis showed a significant correlation between the levels of CRP and fibrinogen (*R*^2^ = 0.376, *P* < 0.001), and the correlation between ASO and age was also significant according to Pearson’s chi-square test (*P* < 0.001) (Additional file 1).

**Table 3:** Univariate and multivariate logistic regression analysis of prognostic factors

Candidate variable	Univariate analysis		Multivariate analysis	
	OR(95% CI)	P value	OR (95% CI)	*P* value
Age ≥ 50 years	0.276 (0.118–0.647)	0.003	0.201 (0.068–0.595)	0.004
Body mass index (kg/m^2^)	1.032 (0.909–1.172)	0.627		
Diabetes mellitus	0.286 (0.053–1.547)	0.146		
Hypertension	0.286 (0.053–1.547)	0.146		
Hyperlipidemia	3.259 (0.351–30.22)	0.298		
Smoker	2.357 (0.831–6.684)	0.107		
CRP > 3 mg/L	0.287 (0.117–0.702)	0.006		
Fibrinogen > 4 g/L	0.172 (0.064–0.459)	< 0.001	0.176 (0.055–0.563)	0.003
Etiology = ASO	0.296 (0.072–1.218)	0.092		
Rutherford class = IV	0.512 (0.151–1.736)	0.283		
Gangrene	0.866 (0.396–1.894)	0.718		
Arterial occlusion above the knee or elbow*	0.249 (0.104–0.595)	0.002	0.232 (0.077–0.703)	0.010
ABI**	8.345 (1.301–53.54)	0.025		
TcPO_2_ (mmHg)	1.069 (1.030–1.109)	< 0.001	1.062 (1.017–1.110)	0.006
Using purified CD34^+^ cells	0.913 (0.425–1.961)	0.816		
Log (total transplanted CD34+ cell counts)***	2.274 (0.850–6.079)	0.102	3.506 (1.021–12.039)	0.046

*Defined as the highest occlusion level located at common iliac artery, external iliac artery, common femoral artery, superficial femoral artery, popliteal artery, axillary artery, or brachial artery

**ABIs of 99 patients with lower limbs treated were included in this analysis, while the other 4 patients with upper limb lesions were excluded

*** Base-10 logarithm of total transplanted CD34^+^ cell counts

OR, odds ratio; CI, confidential interval; CRP, C-reaction protein; ASO, atherosclerosis obliterans; ABI, ankle-brachial index; TcPO_2_: transcutaneous pressure of oxygen; CD, cluster of differentiation

Age ≥ 50 years was chosen for further multivariate regression prior to the etiology (ASO), and the reason is presented in the discussion section. ABI was also removed before the multivariate regression because it could only be examined in the lower limbs (*n* = 99 in our study) and was not suitable for a prognostic analysis of the patients with upper limb ischemia. Either CRP or fibrinogen could be incorporated into the multivariate regression. When incorporating fibrinogen > 4 g/L, the multivariate regression finally indicated that age ≥ 50 years (OR 0.201, 95% CI 0.068–0.595, *P* = 0.004), fibrinogen > 4 g/L (OR 0.176, 95% CI 0.055–0.563, *P* = 0.003), highest arterial occlusion level above the knee or elbow (OR 0.232, 95% CI 0.077–0.703, *P* = 0.010), TcPO_2_ (OR 1.062, 95% CI 1.017–1.110, *P* = 0.006), and Log total transplanted CD34^+^cell count (OR 3.506, 95% CI 1.021–12.039, *P* = 0.046) were independent prognostic factors of responders (Table 3). The regression model was statistically significant (*χ*^2^ = 44.585, *P* < 0.001) and explained 47.1% (Negelkerke *R*^2^) of the variance in the outcome. When incorporating CRP > 3 mg/L, the ORs of the other prognostic factors remained significant, but the Negelkerke *R*^2^ dropped to 42.9% (Additional file 1). Thus, the prior model incorporating fibrinogen > 4 g/L was chosen for the development of the nomogram.

### Nomogram and validation

**Fig. 3:**
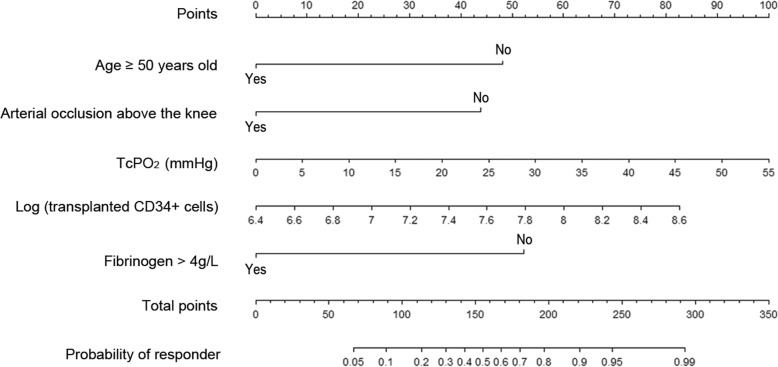
Nomogram predicting responders to mononuclear cell transplantation for no-option critical limb ischemia. Each variable is given a score by projecting its status to the upper point of the scale (0–100) with a straight line. Based on a total score calculated by summing the whole scores of the 5 variables, the predicted probability can be determined by projecting the total score line straight to the probability scale line at the bottom. Log, Base-10 logarithm; TcPO_2_, transcutaneous pressure of oxygen; CD, cluster of differentiation

**Fig. 4:**
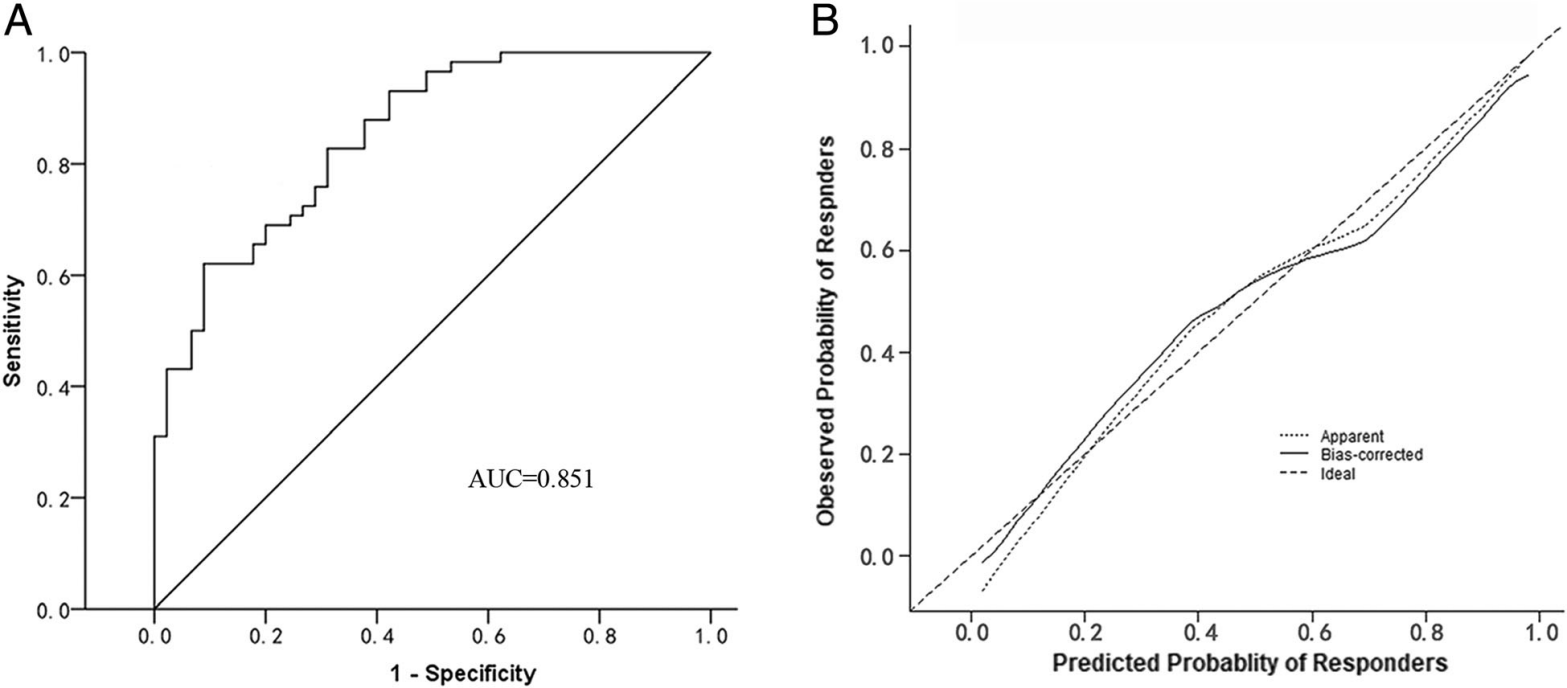
Receiver operating characteristic (ROC) curve to address the sensitivity and specificity of the nomogram, with an area under the curve of 0.851 (**a**) and bias-corrected calibration curve with 1000 cycles of bootstrap resampling to indicate good agreement between the predicted probability and the observed probability of responders (**b**). AUC, area under the curve

**Fig. 5:**
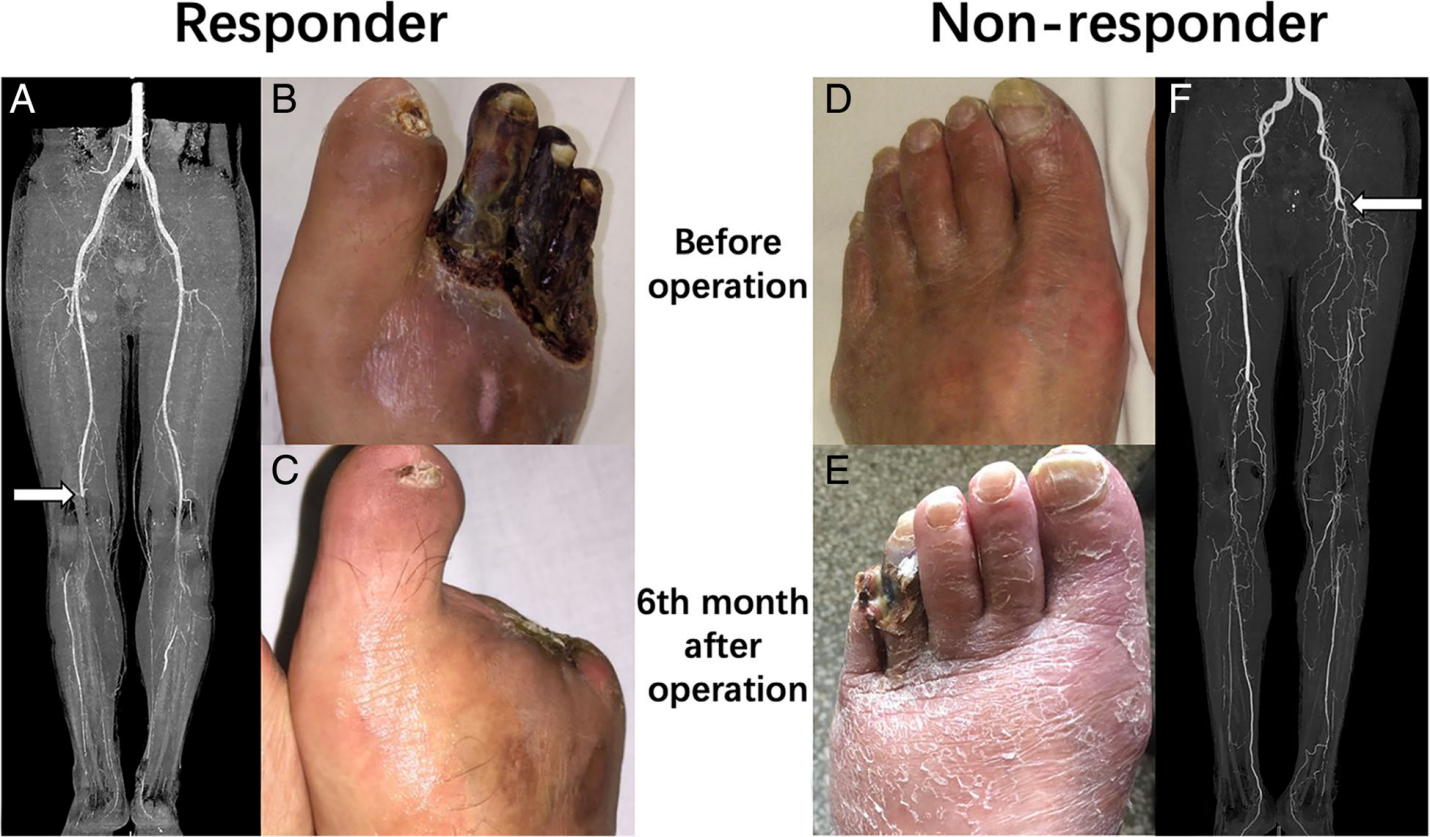
Typical preoperative computed tomographic angiography (CTA) and longitudinal foot photos of a responder (**a**, **b**, **c**) and a non-responder (**d**, **e**, **f**). The case on the left side was a 37-year-old male diagnosed with thromboangiitis obliterans, characterized by progressive limb gangrene (**b**), occlusion level at popliteal artery (**a**), transcutaneous oxygen pressure of 15 mmHg, Log transplanted CD34+ cells of 8.17, and baseline blood fibrinogen < 4 g/L. The nomogram-based score was 192, indicating a probability of 0.76 to be a responder. His toe gangrene was debrided during the transplantation, and the wound healed completely within 6 months (**c**). The case on the right side was a 73-year-old male diagnosed with atherosclerosis obliterans, characterized by left foot rest pain, occlusion level at proximal superficial femoral artery (**f**), transcutaneous oxygen pressure of 12 mmHg, Log transplanted CD34+ cells of 7.38, and baseline blood fibrinogen < 4 g/L. The nomogram-based score was 110, indicating a probability of 0.18 to be a responder. His rest pain was not relieved and the fourth and fifth toes had gangrene at 6 months (**e**). The white arrows indicate the arterial occlusion level

Based on the results of the multivariate regression, a nomogram incorporating five predictors was established to predict the probability of responders (Fig. 3). In the nomogram, each variable was given a score by projecting its status to the upper point scale (0–100) with a straight line. A total score was then calculated by summing up the whole scores of the five variables. The predicted probability of a responder could be determined by projecting the total score line straight to the probability scale line in the bottom (Fig. 3). A ROC curve was generated to address the sensitivity and specificity of the nomogram. The area under the ROC curve was 0.851 (95% CI 0.779–0.922), indicating good discrimination for responders (Fig. 4a). With 1000 cycles of bootstrap resampling, the bias-corrected calibration curve was close to the 45° line, indicating good agreement between the predicted probability and the observed probability of responders (Fig. 4b). The typical CTA and longitudinal foot photos of a responder and a non-responder according to the nomogram were shown in Fig. 5.

## Discussion

### Summary of the results

The current study investigated the prognostic factors related to the therapeutic efficacy of MNC transplantation in treating NO-CLI, and, to our knowledge, this study has initiatively developed a visualized scale to provide an individualized prediction of whether a patient would respond to MNC transplantation. Our results revealed that age ≥ 50 years, blood fibrinogen > 4 g/L, and arterial occlusion above the knee/elbow were negative factors in terms of the probability of being a responder, while the TcPO_2_ value and total transplanted CD34^+^ cell count positively correlated with the probability of being a responder. These prognostic factors were independent from each other, and based on these factors, we built a predictive nomogram with high discrimination and accuracy.

### Prognostic factors associated with patients

Patient age has been considered a critical factor that affects the prognosis after cell therapy. Madaric et al.’s study identified age as an independent predictor for the 1-year amputation-free survival of CLI patients who received MNC transplantation [21]. In our study, we found that age ≥ 50 years was an independent negative prognostic factor for responders who achieved complete CLI remission within 6 months. The critical role of age in the prognosis might be explained with several aspects. First, auto-transplants from elderly patients might not induce sufficient angiogenesis or vasculogenesis due to the aging-related impairment of the survival, migration, differentiation, and paracrine ability of the pro-angiogenic cell fraction [22–24]. Li et al. demonstrated that the ages of human donors correlated inversely with the angiogenic potency of mononuclear cells in preclinical experiments [17]. Accordingly, the aging-related impairment of transplants might explain the inconsistent prognoses of the population at various ages. Second, the relatively poor general condition of elderly patients, complicated by high risks of organ dysfunction or cardiovascular diseases, was likely to reduce the probability of overall survival and amputation-free survival. However, this notion might not explain the age-related CLI remission in our study because the time to endpoint was relatively short and because only one death event occurred, and the enrolled patients were generally in good condition with low frequencies of severe complications. Previous studies have also suggested that the etiology of CLI is a critical prognostic factor. The Therapeutic Angiogenesis by Cell Transplantation (TACT) trial reported that TAO patients who received BM-MNC transplantation had significantly higher major amputation-free rates (1 year 93.2%; 10 years 87.9%) than ASO patients (1 year 78.7%; 10 years 70.1%). The PROVASA trial suggested that TAO patients responded to the MNC therapy more favorably than ASO patients, with a higher probability of rest pain alleviation and ulcer healing [11]. Likewise, our study observed that ASO was associated with a reduced, though insignificant, probability of being a responder. Because the patients with TAO were characterized by a much younger onset age than those with ASO, which was also confirmed in our study, we speculate that age and aging-related cell functional impairments at least partially underlie the relevance of etiology to prognosis. Therefore, age ≥ 50 years was chosen for the multiple regression and nomogram prior to the etiology of ASO, because the former was considered to have a more direct impact on the impaired therapeutic efficacy of MNC transplantation than the latter.

The morphology of the arterial lesion has been considered critical to endovascular and bypass surgery. In contrast, most of the previous clinical studies of cell therapy did not take the anatomical property of the involved arteries into consideration. A recent study, however, found that CLI patients without amputations at 3 months post-MNC transplantation showed more favorable patency of the upper-popliteal arteries than those with major amputations [25]. Our results further indicated that arterial occlusion above the knee or elbow was a significant negative predictor for responders and was independent of age. Notably, in most non-responders (77.8%), the occlusion level involved the popliteal or upper-popliteal artery. We speculate that the above-the-knee patency of the lower limb arteries can bring more favorable blood perfusion to the transplanted site (calf and distal foot), thus enhancing the tissue regeneration induced by cell therapy. Accordingly, we suggest that a sufficient inflow from the upper-popliteal arteries to the calf might be a necessary condition for MNC transplantation. In addition, the significant inverse correlation between the above-the-knee occlusion and responders might partially explain the poor prognosis of cell therapy in ASO patients with CLI because the upper-popliteal arteries, especially the superficial femoral artery, were mostly affected in ASO, while TAO was characterized by the initial invasion of distal small arteries below the knee.

TcPO_2_ represents the tension of oxygen disseminated from subcutaneous microcirculation, which reflects distal peripheral perfusion. The TcPO_2_ measurement has been considered a useful predictor for post-debridement chronic ischemic ulcer healing, with a threshold value of 20–40 mmHg, and a TcPO_2_ value over 40 mmHg seemed to promise complete ulcer healing [26]. In our study, the responders had significantly higher baseline TcPO_2_ values for the distal foot (median 24 mmHg) than the non-responders (median 9 mmHg). The multivariate regression analysis also showed that the baseline TcPO_2_ value was an independent prognostic factor, which was consistent with several previous studies [21, 25]. According to the longitudinal data, we further found that the responders achieved a significant improvement in TcPO_2_ 6 months post-transplantation, while the non-responders did not. Moreover, the TcPO_2_ at baseline correlated significantly with that at 6 months. These results suggested that the restoration of peripheral perfusion induced by MNC transplantation was characterized by a favorable baseline condition of local microcirculation, which might explain the predictive role of baseline TcPO_2_.

Inflammatory biomarkers such as fibrinogen, CRP, and interleukin-6 have recently been considered critical risk factors for the development and progression of PAD [27]. Our study has demonstrated that responders to the MNC transplantation are characterized by significantly lower baseline values of both CRP and fibrinogen than non-responders, and these two biomarkers are significantly correlated with each other according to the linear regression analysis. Because both biomarkers are acute-phase reactant proteins that are produced by the liver during inflammation, we suggest that the systemic or local inflammatory reaction can negatively influence the therapeutic angiogenesis of the MNC transplant. On the one hand, inflammatory factors such as CRP can directly inhibit EPC survival, differentiation, and angiogenic potency [28]. Fibrinogen also plays a leading role in thrombosis, and elevated fibrinogen can increase the cumulative rates of ischemic events in the course of PAD [29, 30]. Our results revealed that both fibrinogen > 4 g/L and CRP > 3 mg/L can be predictors that are independent of the other factors. The former was chosen to develop the nomogram only because it improved the fitting goodness of the model relative to the latter. More studies are required to investigate the most appropriate inflammatory biomarker to predict the prognosis of CLI candidates for cell therapy.

### Prognostic factors associated with the transplants

In addition to the patient’s clinical background, the characteristics of the transplants had a critical influence on the prognosis. CD34^+^ cells represent a main subset of stem/progenitor cells in PBMNC transplants that potentiate neovascularization in the ischemic area [31]. The number of transplanted human CD34^+^ cells appeared to have a strong correlation with the blood perfusion index in the severe combined immunodeficiency mouse model [23]. In addition, the increased number of transplanted CD34^+^ cells improved the chance of wound healing [32]. Previous clinical studies suggested thatCD34^+^cells should be used as quality control for transplants, and a total CD34^+^ cell count over 10^5^/kg body weight seemed appropriate [33]. Our study further demonstrated that the Log total transplanted CD34^+^ cell count was an independent positive predictor of responders, indicating a cell dose-dependent therapeutic efficacy.

On the other hand, we found that responders at 6 months were not associated with transplant purification for CD34^+^ cells or the use of non-purified MNCs. This is consistent with our recent published 12-month results of a prospective randomized trial comparing these two types of cells treating NO-CLI [34]. Accordingly, we believe that both a specific CD34^+^ cell-based and mixed cell-based MNC transplantations might be feasible for NO-CLI patients. A longer-term follow-up study will be needed to compare the advantages and disadvantages of these two transplantation types.

### Feasibility of the nomogram

This originally developed nomogram for predicting complete remissions from NO-CLI at 6 months post-MNC transplantation was based on a consecutive cohort of 110 patients (103 ultimately analyzed) in a single center. The nomogram had a good discriminative ability and predictive accuracy in the validation with the ROC and calibration curves. Notably, our patient population was characterized by a relatively young age and a low frequency of cardiovascular risk and organ dysfunction and was exclusively classified as Rutherford class 4–5. In other studies based on elderly populations with advanced limb ischemia and high risks of systemic diseases, factors such as a history of hemodialysis, Rutherford classification of 6, and high blood glucose also appeared to be relevant to poor prognosis after MNC therapy [16, 35, 36]. Thus, we suggest that our nomogram should not be applied to very elderly individuals with advanced CLI (Rutherford class 6) or a poor general condition to avoid wide uncertainty of the prediction.

### Study limitations

There were several limitations to the current study. First, the current study was retrospectively designed based on a single-centered consecutive cohort, and there was no parallel placebo control group due to ethical concerns. Additionally, the sample size was not large enough for the evaluation of more specific variables, such as cell function and viability. Second, our study focused on short-term responders to MNC transplantation rather than long-term remission from CLI, which is also the target of therapeutic angiogenesis. Therefore, a prolonged follow-up of the cohort is needed in the future to confirm the robustness of the identified prognostic factors. Third, due to the nature of the single-centered design and relatively small sample size of the study, an external validation of the nomogram was not performed, and the generalizability of this predictive tool remains to be examined in cohorts from other centers.

## Conclusion

Age ≥ 50 years, blood fibrinogen > 4 g/L, and arterial occlusion above the knee/elbow were independent negative prognostic factors, while baseline TcPO_2_ and the Log transplanted CD34^+^ cell count were independent positive prognostic factors for responders who survived and achieved complete remission from CLI 6 months post-MNC transplantation. Accordingly, a nomogram with high discrimination and accuracy was developed to provide individualized predictions. A prolonged follow-up study of this cohort is needed to evaluate the robustness of these predictors, and the generalizability of the nomogram remains to be examined in cohorts from other centers.

## Additional file

Additional file 1 Supplementary material. (DOCX 351 kb)

## Abbreviations

ABI: Ankle-brachial index
ASO: Arteriosclerosis obliterans
BM: Bone marrow
CI: Confidence interval
CLI: Critical limb ischemia
IQR: Interquartile range
MNC: Mononuclear cell
NO-CLI: No-option critical limb ischemia
OR: Odds ratio
PAD: Peripheral artery disease
PB: Peripheral blood
ROC: Receiver operating characteristic
SD: Standard deviation
TAO: Thromboangiitis obliterans
TcPO_2_: Transcutaneous pressure of oxygen
