# Supplementary material for: Predictors of responders to mononuclear stem cell-based therapeutic angiogenesis for no-option critical limb ischemia
Source: Stem Cell Res Ther. 2019 Jan 11;10:15. doi: 10.1186/s13287-018-1117-5 (PMC6329149; doi:10.1186/s13287-018-1117-5)
Supplement: Supplementary file 1 — Supplementary material. (DOCX 351 kb) [file 13287_2018_1117_MOESM1_ESM.docx]

**Additional file 1**

**Figures**


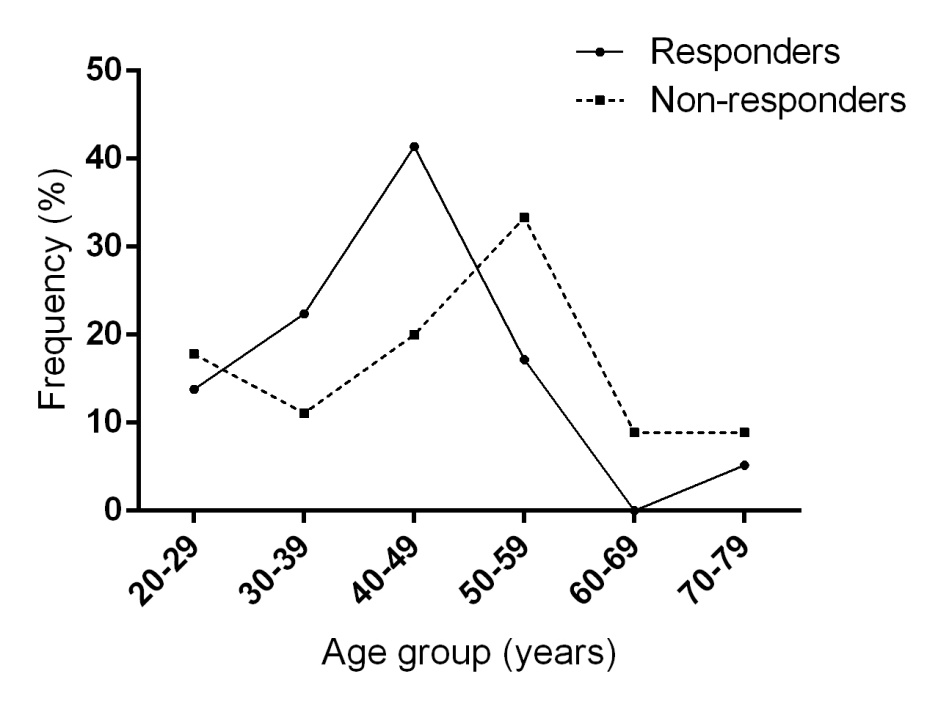


Figure S1. Frequency plots with connecting lines showing the distribution trends of age in the responders and non-responders, respectively.


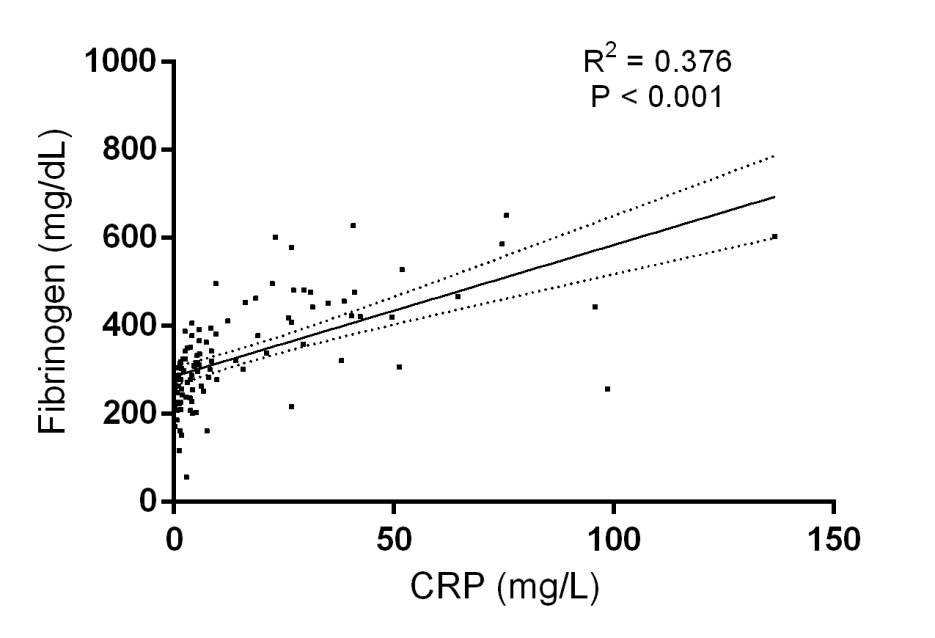


Figure S2. Linear regression plot with the fitting line and standard error bars showing relationship between the value of fibrinogen and CRP (R^2^ = 0.711, P<0.001).

CRP: C-reaction protein


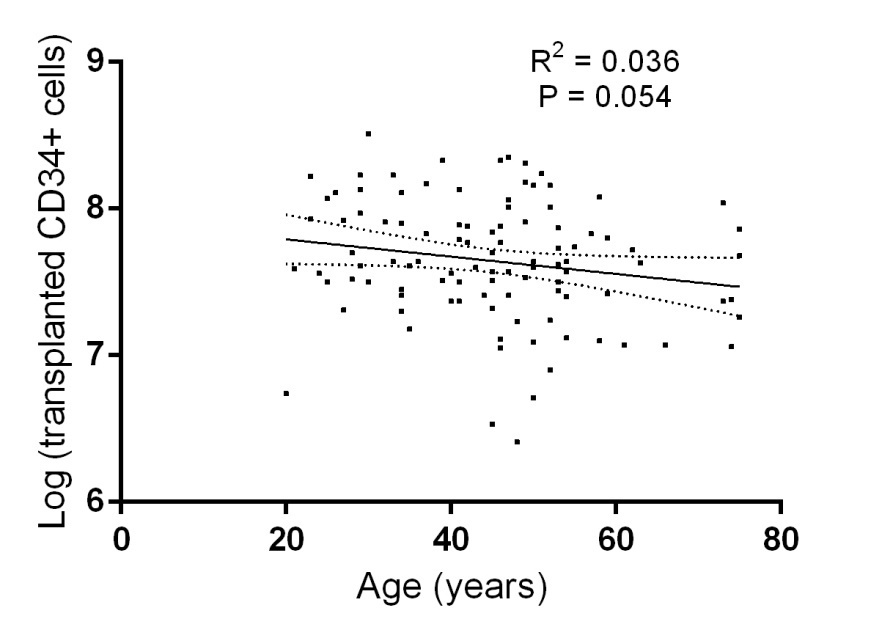


Figure S3. Linear regressionplot with the fitting line and standard error bars showing relationship between the ageand Log (transplanted CD34^+^ cells) (R^2^ = 0.036, P=0.054).


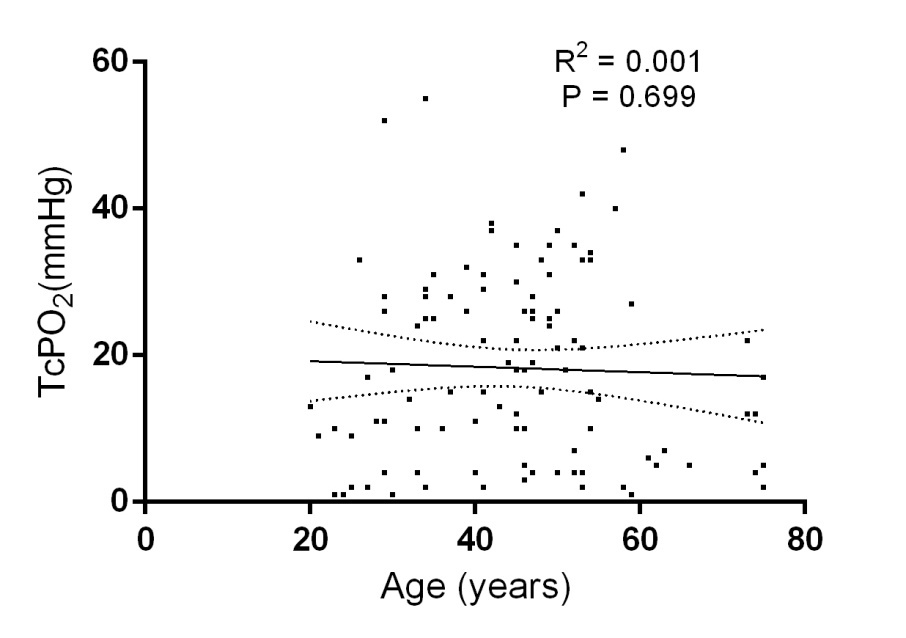


Figure S4. Linear regression plot with the fitting line and standard error bars showing relationship between the ageand TcPO_2_ (R^2^ = 0.001, P=0.699).


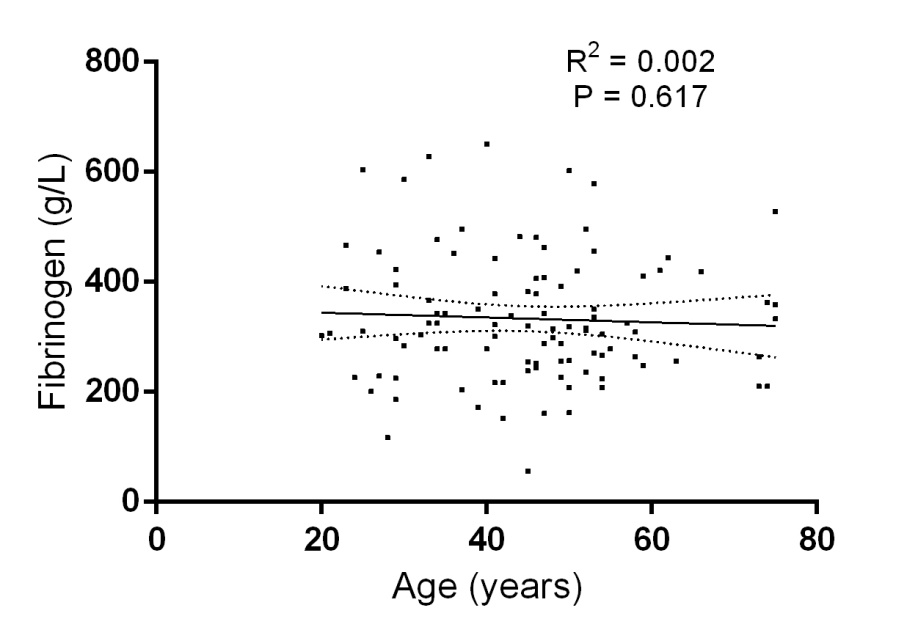


Figure S5. Linear regression plot with the fitting line and standard error bars showing relationship between the ageand fibrinogen (R^2^ = 0.002, P=0.617).

Table S1.Correlation between the age and the etiology of ASO

|  | Etiology=ASO | | χ2 | P value |
| --- | --- | --- | --- | --- |
|  | **Yes** | **No** |  |  |
| Age < 50 years | 0 | 67 | 17.565 | <0.001 |
| Age≥50 years | 10 | 26 |  |  |

ASO, arteriosclerotic obliterans

Table S2.Correlation between the age and the highest level of arterial occlusion

|  | Highest level of arterial occlusion | | χ2 | P value |
| --- | --- | --- | --- | --- |
|  | **above the knee/elbow** | **below the knee/elbow** |  |  |
| Age < 50 years | 41 | 26 | 0.080 | 0.777 |
| Age≥50 years | 21 | 15 |  |  |

Table S3. Multivariate logistic regression incorporating factor of C-reaction protein

| Variable | Multivariate Analysis | |
| --- | --- | --- |
|  | OR (95% CI) | P value |
| Age≥50 years | 0.201 (0.069-0.589) | 0.003 |
| CRP > 3mg/L | 0.293 (0.093-0.922) | 0.036 |
| Arterial occlusion above the knee or elbow* | 0.190 (0.063-0.573) | 0.003 |
| TcPO_2_ (mmHg) | 1.050 (1.007-1.095) | 0.022 |
| Log (total transplanted CD34+ cell counts)** | 3.677 (1.052-12.861) | 0.041 |

* defined as the highest occlusion level located at common iliac artery, external iliac artery, common femoral artery, superficial femoral artery, popliteal artery, axillary artery or brachial artery.

**Base-10 logarithm of total transplanted CD34^+^ cell counts

OR, odds ratio; CI, confidential interval; CRP, C-reaction protein; TcPO_2_: transcutaneous pressure of oxygen; CD, cluster of differentiation
